# Constant direct oral anticoagulants therapy melts away multiple floating thrombi in the aorta

**DOI:** 10.1093/ehjcr/ytaf244

**Published:** 2025-05-16

**Authors:** Ryo Ikeda, Arudo Hiraoka

**Affiliations:** Department of Cardiovascular Surgery, The Sakakibara Heart Institute of Okayama, 2-5-1 Nakaicho, Kita-ku, Okayama 700-0804, Japan; Department of Cardiovascular Surgery, The Sakakibara Heart Institute of Okayama, 2-5-1 Nakaicho, Kita-ku, Okayama 700-0804, Japan

**Figure:**
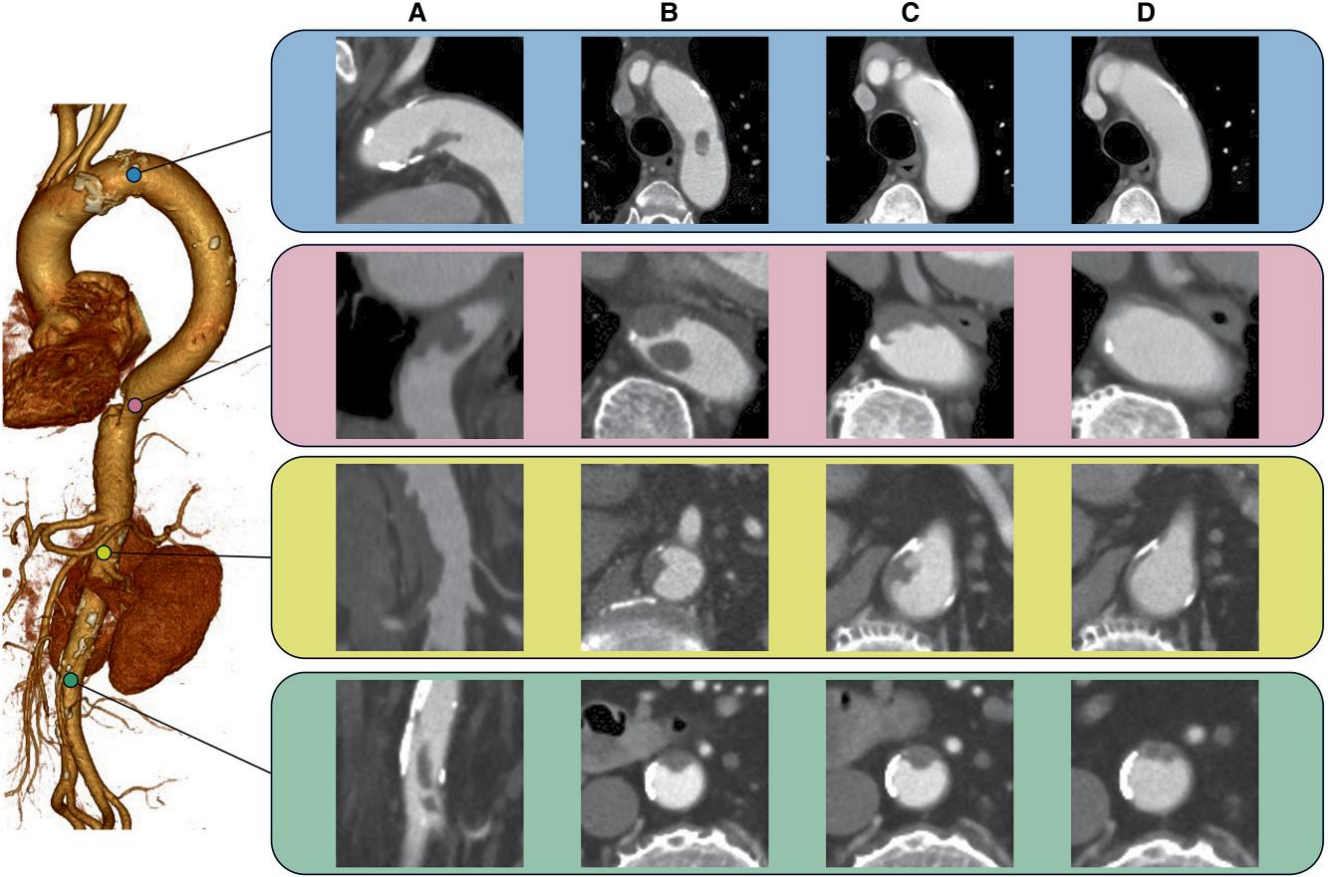


An 89-year-old man presented to the emergency department with abdominal pain. Computed tomography showed multiple aortic floating thrombi (MAFT) (*Panels A* and *B*) and transthoracic echo showed a floating thrombus in the descending aorta (see *Supplementary material online, Video S1*). Laboratory tests showed a platelet count of 120 × 10^4^/μL and bone marrow aspiration revealed primary myelofibrosis leading to abnormal thrombocytosis. Direct oral anticoagulants (DOACs) and antiplatelet agents were administered for MAFT and thrombocytosis. We followed the process of MAFT resolution for 4 weeks (*Panel C*) and 8 weeks (*Panel D*) without visceral branches and peripheral arterial emboli. MAFT is a rare disease that can be catastrophic due to visceral branches and peripheral arterial embolism.

The aetiology of MAFT is often unclear, however many of the cases have been associated with atherosclerosis of the aorta and abnormal coagulation function is reported to be one of the risk factors for MAFT.^1^ There are several reports of surgical thrombectomy and TEVAR for aortic floating thrombus,^2,3^ which are highly invasive and can result in the spread of thrombus to the visceral branches and lower limbs. In addition, these procedures for MAFT have a risk of spinal code injury. On the other hand, there are only a few reports on the conservative treatment of MAFT with DOACs. We chose a DOAC instead of warfarin or LMWH, since DOACs have a rapid onset of therapeutic effect and do not require dose control until they are effective. Additionally, DOACs are also superior in terms of adherence to long-term anticoagulation therapy. We have successfully managed MAFT conservatively with DOACs, which can be a useful option to avoid excessive invasiveness and complications when the aortic condition is not abnormal and the culprit disease is known and treatable.

## Supplementary Material

ytaf244_Supplementary_Data

## Data Availability

The data underlying this article are available in the article and in its online Supplementary material.

## Notes

**Consent:** The authors confirm that written consent for submission and publication of this manuscript including the images and associated text has been obtained from the patient in line with the COPE guidelines.
